# Understanding the Gap Between Nutritional Knowledge and Dietary Behavior Among Adolescents with Different BMI Statuses

**DOI:** 10.3390/nu18142287

**Published:** 2026-07-13

**Authors:** Agata Wawrzyniak, Iwona Traczyk

**Affiliations:** 1 Department of Human Nutrition, Institute of Human Nutrition Sciences, Warsaw University of Life Sciences (SGGW-WULS), 02-787 Warsaw, Poland; 2 The Department of Environmental Hazard Prevention, Allergology and Immunology, Faculty of Health Sciences, Medical University of Warsaw, 02-007 Warsaw, Poland; iwona.traczyk@wum.edu.pl

**Keywords:** nutrition-related knowledge (NRK), nutrition-related practice (NRP), nutrition literacy, food literacy, adolescents, body mass index (BMI), physical activity, socio-demographic factors

## Abstract

**Background/Objectives**: The aim of this study was to examine the discrepancy between nutrition-related knowledge (NRK) and nutrition-related practice (NRP) among 1440 Polish school-aged students (10–18 years), according to BMI status, and to identify factors associated with this discrepancy. **Methods**: This cross-sectional study was conducted via the CAWI method using an author-developed, non-validated questionnaire. Questions assessing NRK and NRP were thematically aligned and referred to the recommendations of the Polish Healthy Eating and Lifestyle Pyramid for Children and Adolescents (aged 4–18 years). **Results**: Students with excess weight scored significantly lower in nutritional knowledge (NRK: 46%) and practices (NRP: 30%) than those with underweight or normal weight (NRK: 52–53%, NRP: 33–34%). Higher NRK was associated with older age, female sex, larger urban residence (for students with overweight and obesity), and higher maternal/legal guardian education level (for students with normal/underweight body weight). Better NRP was associated with higher NRK and higher physical activity, while rural or small-town living was positively associated with healthier dietary practices and maintenance of normal BMI. **Conclusions**: The present study confirms that nutritional knowledge is a necessary but insufficient condition for shaping appropriate dietary behaviors among adolescents with different BMI statuses; lifestyle factors, practical food-related skills—including self-assessment of knowledge and adherence to dietary recommendations—and the broader environmental context plays a crucial role. These findings highlight the need for targeted nutrition education programs that expand not only knowledge but also practical skills among students.

## 1. Introduction

Adolescence is recognized by the World Health Organization as a critical period for the development of health-related behaviors, including dietary habits and patterns of physical activity, which largely determine health status later in life [1,2]. The WHO emphasizes that adequate nutrition during this stage is fundamental for optimal growth, maturation, cognitive development, and metabolic programming, and constitutes one of the key elements in the prevention of non-communicable diseases in adulthood [1,2]. At the same time, according to current WHO data, the global adolescent population is facing the so-called double burden of malnutrition, encompassing both nutritional deficiencies and the growing prevalence of overweight and obesity. This phenomenon makes adolescent nutrition one of the major priorities of contemporary public health [1,3]. Current research indicates that unhealthy dietary patterns and insufficient physical activity are among the key factors contributing to abnormalities in nutritional status among adolescents [4,5,6].

Current research indicates that unhealthy dietary patterns and insufficient physical activity are among the key factors contributing to abnormalities in nutritional status among adolescents [4,5,6].

Dietary guidelines for children and adolescents emphasize the importance of regular meal consumption; adequate intake of vegetables and fruits, whole-grain products, milk and dairy products, and fish; as well as the need to limit foods high in added sugars, salt, and saturated fats [7]. Despite the widespread availability of these recommendations and the implementation of numerous health education programs [8,9,10,11,12], adherence to healthy eating principles among adolescents remains unsatisfactory in many countries [2,5,12].

Nutritional knowledge is widely recognized as one of the key prerequisites for the development of appropriate dietary behaviors. Recent population-based studies confirm that adolescents’ nutritional knowledge is most often moderate and uneven, with significant gaps related, among others, to the role of specific dietary components and principles of proper meal planning [2,6]. Systematic reviews and observational studies further indicate that the relationship between nutritional knowledge and actual dietary behaviors in children and adolescents is often weak or inconsistent [13,14]. Earlier studies have already shown that adolescents may demonstrate a relatively good level of nutritional knowledge while simultaneously maintaining unhealthy dietary habits [14]. This phenomenon is referred to as the “knowledge–practice gap” and represents one of the major challenges of contemporary nutrition education [13,14].

More recent evidence suggests that the knowledge–practice gap should be interpreted within a broader behavioral framework. Adolescents’ food choices are influenced not only by nutritional knowledge but also by self-regulation, family and peer influences, and the rapidly evolving digital food environment, including social media and online food marketing [15,16,17]. Consequently, nutrition education should extend beyond knowledge transfer to strengthen food literacy and digital health literacy [18].

Well-designed nutrition education programs can improve both nutritional knowledge and diet quality; however, achieving sustained behavioral change requires an approach that goes beyond the mere transmission of information [15,19]. Increasing attention is therefore being paid to the development of so-called nutrition (food) literacy, which encompasses not only knowledge, but also practical skills, motivation, and the capacity for self-regulation [15,20].

The relationship between nutritional knowledge and nutritional status assessed using body mass index (BMI) remains complex and inconclusive. Some population-based studies have shown that higher levels of nutritional knowledge are associated with a lower risk of being overweight and of obesity among children and adolescents [5,21]. Conversely, both earlier and more recent studies suggest that excess body weight may coexist with relatively good theoretical knowledge that is not reflected in everyday dietary choices [4,14,15]. Despite the growing body of literature on adolescent nutrition, relatively few population-based studies have simultaneously examined nutritional knowledge, dietary behaviors, and sociodemographic and lifestyle determinants across groups with different BMI status [4,5,21]. Therefore, the aim of the present study was to assess nutritional knowledge and adherence to nutritional recommendations among school-aged youth according to nutritional status (BMI), and to identify sociodemographic and lifestyle factors associated with both the level of knowledge and its practical application. Particular emphasis was placed on the knowledge–practice gap and the role of physical activity as a key determinant of health-related behaviors. Given the increasing complexity of adolescents’ food environments, understanding the relationship between nutritional knowledge and its practical application remains an important public health challenge. To our knowledge, this is the first population-based study in Poland using thematically paired NRK–NRP questions to quantify the knowledge–behavior gap across BMI categories.

## 2. Materials and Methods

### 2.1. General Information

An anonymous and voluntary cross-sectional study was conducted using a computer-assisted web interviewing (CAWI) method among 1440 Polish school-aged students. The study protocol was approved by the Bioethics Committee of the Institute of Human Nutrition Sciences, Warsaw University of Life Sciences (approval No. 18/2021, dated 1 June 2021) and was conducted in accordance with the principles of the Declaration of Helsinki.

### 2.2. Participant Recruitment

The recruitment of students and schools was conducted via social media platforms (i.e., Facebook and Twitter). Detailed information on the recruitment procedure was provided in our previous publication [4]. The inclusion criteria were as follows: student consent; parental/legal guardian informed consent for the student’s participation in the study; consent for personal data processing; student age between 10 and 18 years; and attendance at schools that had agreed to participate in the study. The survey was conducted during school classes in computer laboratories. Each student completed the questionnaire individually using the Google Forms application. A post hoc analysis based on the specified parameters (power of 80% and significance level of 5%) showed that the minimum required sample size for the study was 950 participants. The study group met the required size criteria.

A full characterization of the study sample was published in an article by Wawrzyniak and Traczyk [4].

### 2.3. Questionnaire Survey

An original questionnaire for the assessment of nutrition-related knowledge (NRK) and nutrition-related practices (NRP) was developed for the purpose of the study [4]. The main part of the survey consisted of two thematic sections, each comprising 12 questions assessing NRK or NRP, respectively, which were thematically paired. Questions related to NRK and NRP referred to the recommendations and principles of the Polish Healthy Eating and Lifestyle Pyramid for Children and Adolescents (aged 4–18 years), published by the National Institute of Public Health–National Institute of Hygiene [7].

In a separate section of the questionnaire, students provided information on sex, age, place of residence (rural area, town with up to 50,000 inhabitants, city with more than 50,000 inhabitants), maternal/legal guardian educational level (primary, secondary, higher), level of physical activity (low—predominantly sedentary leisure time; moderate—approximately equal amounts of sedentary and active time; high—predominantly active leisure time; based on [22]), self-assessed nutritional knowledge, and self-assessed adherence to dietary recommendations (insufficient, sufficient, good, very good). All questions were single choice. Knowledge-related questions included the response option “I do not know”. Additionally, the questionnaire included open-ended questions on respondents’ height and body weight, which were self-reported and verified with parents/legal guardians.

### 2.4. Assessment of Students’ NRK and NRP

The assessment of students’ nutritional knowledge (NRK) and nutritional practices (NRP) was based on the scores obtained from the questionnaire [4]. For each correct answer, in accordance with the recommendations of the Polish Healthy Eating and Lifestyle Pyramid for Children and Adolescents [7], one point was awarded; incorrect answers or the response option “I do not know” were assigned zero points. The maximum possible score for both the NRK and NRP indices was 12 points. Based on the total number of points obtained, students were classified into the following categories: insufficient (0–3 points), sufficient (4–6 points), good (7–9 points), and very good (10–12 points).

### 2.5. Assessment of Nutritional Status

Students’ nutritional status was assessed by calculating body mass index (BMI) based on self-reported body weight and height [4]. BMI values were compared with age- and sex-specific BMI percentile charts for the Polish population to classify nutritional status into the following categories: underweight, normal weight, overweight, and obesity, in accordance with established guidelines [23].

### 2.6. Statistical Analysis

Statistical analyses were performed using the IBM SPSS Statistics version 29 (SPSS Inc., Chicago, IL, USA). The Shapiro–Wilk test was used to assess data distribution. The variables showed a skewed distribution. Depending on the type of variable, results were expressed as mean, standard deviation, median, range, or percentage. BMI categories—underweight, normal weight, and excess body weight (i.e., overweight/obesity)—were used as the differentiating factor in the assessment of NRK and NRP. Statistical significance between subgroups was evaluated using Pearson’s chi-square test (for categorical variables expressed as percentages) and the Kruskal–Walliss test (for continuous variables). Additionally, the Wilcoxon signed-rank test was applied to compare paired results. The Wald test was used to evaluate the associations. Students with normal BMI served as the reference group (OR = 1). The Wilcoxon effect size (r) was calculated for the differences between NRK and NRP. Effect sizes were interpreted as small (r < 0.30), medium (0.30 ≤ r < 0.50), or large (r ≥ 0.50). The association between nutritional knowledge and adherence to dietary recommendations was examined using Spearman’s rank correlation coefficient. Linear regression models were tested to evaluate determinants of NRK and NRP associated with sociodemographic and lifestyle factors across BMI categories (underweight, normal weight, and excess body weight, i.e., overweight/obesity). A *p*-value ≤ 0.05 was considered statistically significant.

## 3. Results

### 3.1. Characteristics of the Student Sample According to BMI

Among the 1440 students aged 10–18 years, 11% were underweight, 70% had normal weight, and 19% were overweight or in the obesity category (Table 1).

The distribution of sex differed across BMI categories. Girls predominated among students with underweight (76%); whereas, boys predominated among those who were overweight or experienced obesity (71%). Students who were overweight or experienced obesity were roughly 0.5 years younger than peers. Maternal/legal guardians’ primary education was more common among underweight and overweight/obese groups (14–16% vs. 10% normal); whereas, higher education was most common in the normal-weight group (59%). Students with underweight rated both their nutritional knowledge and dietary practices more favorably (very good/good: 66% and 81%, respectively) compared with students with excess body weight (very good/good: 51% and 70%, respectively) and those with normal body weight (very good/good: 56% and 79%, respectively). Place of residence and physical activity levels did not differ by BMI group. In the study population 33–37% lived in rural areas, 17–25% reported low physical activity, and 42–54% reported moderate activity.

### 3.2. Students’ NRK and NRP According to BMI

**Figure 1:**
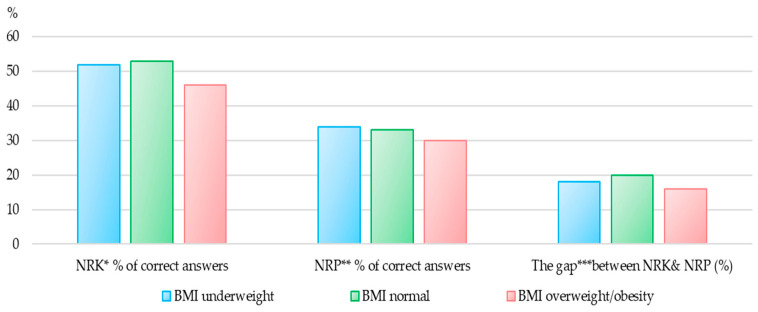
Assessment of students’ NRK and NRP, and the gap between them according to BMI (N = 1440). * Kruskal–Wallis test (*p* < 0.001); ** Kruskal–Wallis test (*p* = 0.007); *** Wilcoxon test (*p* < 0.001).

NRK scores were significantly higher than NRP scores across all BMI categories (Figure 1). Students with underweight or normal weight achieved NRK scores of 52–53%; whereas, their NRP scores reached only 33–34%. Students with excess body weight scored lower, averaging 46% for knowledge and only 30% for practice.

Compared with students with normal weight, those who were overweight or obese had significantly lower NRK scores. Students with excess body weight performed worse on questions considered key to maintaining a healthy body weight, including: “How many meals should children and adolescents eat per day?”, “Which regularly consumed meal is particularly important for well-being at school?”, “How often should children and adolescents eat fruit and vegetables?”, “Which products contain more dietary fiber?”, “Which type of meat and/or meat products is most recommended for consumption?”, “Which products should replace sweets?”, and “Which products contain a lot of salt?”. The nutritional knowledge of students who were underweight differed significantly (and was lower) compared with students with normal body weight only for two questions: “How often should children and adolescents eat sea fish?” and “Which products contain a lot of salt?” (Table 2).

Similarly, the dietary practices (NRP) of students with excess body weight differed significantly from those of students with normal body weight. A higher number of irregularities were identified in responses to the following items: “Do you eat breakfast in the morning before going to school?”, “How often do you eat fruit and vegetables?”, and “Which type of meat and/or meat products do you eat most often?”. In contrast, the dietary practices of students with underweight did not differ significantly from those of students with normal body weight, with the exception of a higher level of self-reported physical activity.

In the NRK assessment, students achieved less than 20% correct responses for questions related to milk consumption (16–18%) and sea fish intake (9–22%). In the NRP assessment, regardless of BMI category, only up to 20% of respondents reported dietary behaviors consistent with recommendations for the consumption of whole-grain cereal products (12–18%), milk and dairy products (11–15%), fish (7–8%), sweets (3–5%), and sweet snacks (4–8%).

### 3.3. Association Between Nutritional Knowledge and Dietary Practices According to Students’ BMI

In each of the studied groups, a statistically significant association between nutritional knowledge (NRK) and dietary practices (NRP) was observed; this relationship was strongest among students with normal body weight (r = 0.376, r-Wilcoxon effect size = 0.73) (Table 2 and Table 3). In all BMI groups, a significant association was also found between self-assessed nutritional knowledge and self-assessed adherence to dietary recommendations. In groups with abnormal body weight (underweight and excess body weight), no significant association was observed between students’ self-declared nutritional knowledge and researcher-assessed NRK. In contrast, such an association was identified for the implementation of dietary practices, with the strongest relationship observed among students with normal body weight (r = 0.304). With the exception of self-assessed nutritional knowledge among students with normal body weight, participants were generally unable to accurately assess their own nutritional knowledge and adherence to dietary recommendations, substantially overestimating their self-assessed compliance with dietary guidelines (Table 1).

### 3.4. Sociodemographic and Lifestyle Factors Determining NRK and NRP According to BMI

It was estimated that students’ nutritional knowledge (NRK), regardless of BMI category, was significantly and positively associated with age (with knowledge increasing with age) and sex (higher among girls). Among students who were overweight and obese, place of residence (larger urban areas) was a significant determinant of higher NRK scores; whereas, among students with underweight and normal body weight, higher maternal/legal guardian educational attainment was associated with better NRK outcomes (Table 4).

With regard to determinants of dietary practices (NRP), a positive and statistically significant association with NRK and physical activity was observed, regardless of BMI status (*p* < 0.0001). Living in rural areas or smaller towns was conducive to better adherence to dietary recommendations and the maintenance of normal body weight. No significant effects of other sociodemographic factors on NRP were identified among students.

## 4. Discussion

The results of the present study clearly indicate the existence of a substantial gap between the level of nutritional knowledge (NRK) and the actual implementation of nutrition-related practice (NRP) among school-aged adolescents, regardless of BMI status. Across all analyzed groups, the proportion of correct responses reflecting nutritional knowledge was significantly higher than the level of adherence to dietary recommendations in practice. This finding confirms that nutritional knowledge alone does not automatically translate into healthy dietary behaviors. Importantly, among adolescents with excess body weight, both NRK and NRP were assessed at significantly lower levels compared with their peers with normal body weight. This phenomenon has been widely described in the literature as the “knowledge–behavior gap” and represents one of the major challenges of contemporary nutrition education [4,13,14,15]. The present findings are consistent with our earlier reports, which demonstrated a moderate level of nutritional knowledge among adolescents accompanied by a significantly poorer implementation of dietary recommendations in everyday practice [4]. Similar conclusions emerge from the systematic review by Thakur and Mathur [13], who demonstrated that in the majority of studies the relationship between nutritional knowledge and dietary behaviors among children and adolescents was weak or non-significant. Likewise, the study by Naeeni et al. [14] confirmed that students may report good nutritional knowledge while simultaneously maintaining unhealthy dietary habits.

In recent years, increasing attention has been given to the concept of nutrition/food literacy as a key framework for explaining this discrepancy, as it encompasses not only knowledge, but also practical competencies and the capacity for self-regulation [24,25,26]. Recent evidence suggests that the knowledge–practice gap should be interpreted within a broader behavioral framework rather than as a simple consequence of insufficient nutrition education [15,18]. Contemporary behavioral nutrition models indicate that adolescents’ food choices result from complex cognitive, social, and environmental influences rather than nutritional knowledge alone [15,16,27]. During adolescence, environmental and social influences may outweigh nutritional knowledge when making everyday food choices [15,16]. The rapidly evolving digital food environment represents another important mechanism explaining why nutritional knowledge is often insufficient to promote healthy eating behaviors. Adolescents are continuously exposed to food-related content through social media, influencer marketing, online food delivery services, and digital advertising of highly processed foods. These influences may normalize energy-dense, nutrient-poor diets and make them appear socially desirable. Consequently, environmental cues may override cognitive intentions, making it more difficult to translate nutritional knowledge into healthy dietary practices [27,28].

### 4.1. The Impact of Nutritional Status (BMI) on NRK and NRP Levels Among Students

An important finding of the present study is the demonstration that adolescents with excess body weight were characterized by both a lower level of nutritional knowledge and poorer adherence to dietary recommendations compared with their peers with normal body weight and even compared with those who were underweight. This result is consistent with the findings of Iyassu et al. [5], who showed that nutritional knowledge influences dietary behaviors and indirectly affects the risk of overweight and obesity, as well as with the study by Wang et al. [21], who reported that higher levels of nutritional knowledge were associated with a lower risk of excess body weight among children and adolescents. At the same time, both our results and existing literature indicate that excess body weight may coexist with relatively good theoretical knowledge that is not reflected in everyday dietary choices [4,6,14]. This underscores the crucial role of behavioral, motivational, and environmental factors in shaping nutritional status. Accordingly, excess body weight should be viewed not merely as a consequence of insufficient nutritional knowledge but as the result of repeated food-related decisions shaped by complex family, school, social, economic, and digital environments [16,27]. Cognitive psychology describes optimism bias, overconfidence, and illusory superiority as mechanisms through which individuals tend to evaluate their behaviors more favorably than objective evidence would justify [29,30].

In the context of nutrition, adolescents may compare their eating habits with those of their peers rather than with evidence-based dietary recommendations. Consequently, when unhealthy dietary practices are common within the social environment, they may be perceived as normal and acceptable. This may lead adolescents to overestimate the quality of their own diets despite objectively inadequate eating behaviors [16].

Moreover, research on body weight perception and body image suggests that inaccurate self-assessment promotes the persistence of unfavorable health-related behaviors [31,32,33], which aligns well with our observations regarding the limited accuracy of self-assessed nutritional knowledge and the lower accuracy of self-reported adherence to dietary recommendations among students with abnormal BMI. Distorted body and weight perception may reduce motivation to modify unhealthy dietary behaviors and delay recognition of obesity-related risk [31,32,33,34].

From a practical perspective, inaccurate self-assessment represents one of the greatest barriers to successful nutrition education [15,34]. Adolescents who perceive their dietary habits as appropriate are less likely to recognize unhealthy behaviors, seek nutritional guidance, or engage in behavior change [16,34]. Therefore, nutrition education should incorporate self-monitoring, individualized feedback, and opportunities for critical reflection that help adolescents compare their dietary behaviors with evidence-based recommendations rather than with subjective perceptions or peer norms [15,18]. Such approaches are particularly important in adolescence, when body image, social comparison, and peer acceptance play a major role in self-evaluation and health-related decision-making [31,32,33,34].

Particularly noteworthy, regardless of BMI status, are the low or very low levels of adherence to recommendations for the consumption of vegetables and fruits, whole-grain products, milk and dairy products, and fish, alongside a high risk of excessive intake of sweets and salty snacks. This pattern was observed despite often better nutritional knowledge in these areas, as seen for vegetables and fruits, whole-grain products, and sweet and salty snacks. This phenomenon is consistent with European and global data indicating persistently poor diet quality among adolescents—especially those with higher BMI—and unfavorable trends in the consumption of vegetables, fruits, and foods of high nutritional value [35,36,37], accompanied by excessive intake of foods of low nutritional value [33] and irregular breakfast consumption [38]. These issues warrant particular attention and were also confirmed by the present study. Our findings indicate that irregularities in the consumption of vegetables and fruits, meat and meat products as sources of saturated fatty acids and skipping breakfast may be considered key factors contributing to excess body weight. These associations have also been reported in previous studies [33,35,36,37,38]. However, these behaviors should also be interpreted within the context of food availability, affordability, family meal patterns, and peer norms, all of which may limit the adoption of healthy dietary behaviors despite adequate nutritional knowledge [2,16,39].

### 4.2. Evaluation of Sociodemographic and Lifestyle Factors Determining NRK and NRP According to BMI

Adolescents’ dietary behaviors are shaped by a complex interaction of individual, familial, and environmental factors. Among these, sociodemographic variables—such as sex, age, and parental educational level—as well as lifestyle—related factors, particularly the level of physical activity, play an important role [4,5,15]. Previous studies have also indicated a significant influence of the family environment and parents’ nutritional knowledge on children’s dietary habits [40]. However, family influences extend beyond parental education and include parental modeling, household food availability, and economic access to healthy food [16,40]. Peer influence also becomes increasingly important during adolescence, as eating behaviors often reflect social identity and group belonging rather than individual nutritional preferences alone [39]. Consistent with earlier reports [4,6], girls and older students exhibited higher levels of nutritional knowledge (NRK), regardless of BMI status, in the present study. Family environment, particularly the educational level of the mother/legal guardian, had a significant impact on NRK [40]. However, these factors did not translate into a correspondingly better implementation of dietary recommendations, once again confirming the existence of a gap between knowledge and practice. This result suggests that sociodemographic advantages may improve awareness but do not necessarily equip adolescents with practical food literacy skills, self-regulation, or supportive environments needed to adopt healthy dietary behaviors [15,16].

Adolescents living in rural areas also demonstrated better adherence to dietary recommendations than their urban counterparts. Although the present study was not designed to identify the underlying causes of this difference, several explanations appear plausible. Rural environments may facilitate healthier eating through greater access to locally produced foods and more frequent family meal preparation; whereas, urban environments may expose adolescents to highly processed foods and digital food marketing more frequently. These findings suggest that characteristics of the local food environment may influence adolescents’ dietary behaviors independently of nutritional knowledge. Future longitudinal studies should investigate which environmental factors are most strongly associated with healthier dietary patterns in rural and urban settings [2,16].

One of the strongest predictors of appropriate adherence to dietary recommendations (NRP) in our study was the level of physical activity. Students who were more physically active were significantly more likely to follow healthy eating principles, regardless of BMI category. This finding is consistent with the concept of “health behavior clustering” and with population-based studies demonstrating a strong association between physical activity and diet quality among adolescents [4,5,41,42]. From a public health perspective, this suggests that promoting physical activity may serve as an effective entry point for improving other lifestyle components, including dietary habits. School-based sports and physical education programs may provide an ideal platform for integrating practical nutrition education into everyday school life. Activities such as meal planning, food label reading, healthy snack preparation, and cooking workshops may strengthen adolescents’ practical food literacy skills and facilitate the adoption of healthy dietary behaviors. Integrated interventions are likely to be more effective than traditional classroom-based nutrition education delivered in isolation [15,18].

Moreover, contemporary theoretical and empirical frameworks emphasize that effective and sustained changes in dietary behavior require the development of broadly defined nutrition competencies, including meal planning, cooking skills, food label literacy, and self-regulation [24,25,26]. Importantly, food literacy extends beyond nutritional knowledge and includes practical skills such as shopping, cooking, and food label reading. It also involves the ability to critically evaluate nutrition information from digital media and social networks, an increasingly important competency for adolescents navigating today’s digital food environment [18,43]. Research indicates that higher levels of nutrition/food literacy are associated with better diet quality among children and adolescents [25,26,44], as well as with improved diet quality and higher physical activity levels among university students [45]. Moreover, nutrition literacy has been shown to mediate the relationship between lifestyle factors and healthy dietary behaviors, highlighting the gap between nutritional knowledge and practical food-related skills [45]. This helps explain why, in the present study, higher theoretical knowledge alone was insufficient to achieve a comparably high level of adherence to dietary recommendations, despite the observed association between NRK and NRP. At the same time, systematic reviews and intervention studies demonstrate that while educational programs can improve nutritional knowledge, the sustainability of behavioral change depends largely on their comprehensiveness and integration into students’ everyday environments [12,19,46]. Particularly favorable outcomes have been reported for interventions combining nutrition education with physical activity promotion and practical skills training [12,19,47].

From a public health perspective, these findings suggest that effective nutrition interventions should move beyond traditional information-based education. Greater emphasis should be placed on strengthening food literacy, self-regulation, and practical food-related skills. Such multidimensional approaches are likely to achieve more sustainable improvements in adolescents’ dietary behaviors than programs focusing solely on increasing nutritional knowledge [15,18].

The present findings are consistent with current recommendations of the World Health Organization, which emphasize the need for an integrated approach to adolescent nutrition that combines education, physical activity promotion, and modification of the living environment [1,48]. In practice, this implies a shift away from narrowly defined, information-based education toward long-term, environment-oriented strategies for changing dietary behaviors.

Future obesity prevention programs should integrate nutrition education with practical food-related skills, physical activity promotion, and supportive family and school environments. Such integrated approaches are likely to support more sustainable improvements in adolescents’ dietary behaviors [15,18]. Future educational programs should strengthen adolescents’ awareness of their own dietary behaviors. Recognizing discrepancies between perceived and actual eating habits appears to be a prerequisite for sustainable behavioral change.

### 4.3. Strengths and Limitations of the Study

The present study has several notable strengths. It is a pioneering study in Poland that assesses the discrepancy between nutritional knowledge and the implementation of dietary recommendations—using thematically paired questions addressing knowledge and practice—among students with different BMI statuses. An important contribution of this research is the precise identification of specific areas of nutritional knowledge and self-reported dietary practices that require improvement or correction across BMI categories. The study was conducted in a large sample (N = 1440) of Polish adolescents spanning a wide age range (10–18 years) and included a broad set of sociodemographic variables (age, sex, place of residence, and maternal/legal guardian educational level), as well as physical activity and BMI. This comprehensive approach is particularly valuable for the development of personalized nutrition education programs aimed at improving BMI and overall health, as it enables the identification of both the gap between NRK and NRP and the factors determining nutritional knowledge and adherence to dietary recommendations depending on BMI status. An additional strength of the study is the comparison of objectively assessed NRK and NRP with students’ self-assessments among those with abnormal body weight.

The study has several limitations. The questionnaire used to assess nutritional knowledge and practices was developed specifically for this study and was not formally psychometrically validated, which may have affected measurement accuracy. However, a pilot study involving 30 students of different ages was conducted before data collection to ensure that the questions were clear and consistent with the recommendations of the Polish Healthy Eating and Lifestyle Pyramid for Children and Adolescents, as described previously [4]. Participant recruitment via social media and voluntary participation may have resulted in selection bias and limited representativeness of the broader population of Polish youth. Dietary practices and physical activity were assessed based on self-reported data, increasing the risk of recall and social desirability biases. Body weight and height were also self-reported, which could lead to the misclassification of BMI categories, although the literature confirms a high correlation between self-reported data and actual measurements [49]. Potentially important determinants of dietary behavior, such as household income, the food environment, screen time, social media exposure, psychological factors, and parental dietary behaviors, were not evaluated. Although this study identified factors associated with NRK and NPR, its cross-sectional design and reliance on self-reported data preclude causal inferences regarding the relationship between nutritional knowledge and dietary practices.

## 5. Conclusions

The present study confirms that nutritional knowledge is a necessary but insufficient condition for the development of appropriate dietary behaviors among adolescents; lifestyle factors, practical competencies, and the environmental context play a crucial role. Both nutritional knowledge and adherence to dietary recommendations were significantly lower among students with excess body weight. Regardless of BMI status, higher NRK was associated with age and female sex (higher among girls); whereas, NRP was dependent on NRK and physical activity levels. Among students with overweight and obesity, higher NRK scores were associated with residence in larger urban areas, while among students with underweight and normal body weight, higher maternal/legal guardian educational attainment was a significant determinant of NRK. Dietary practices were additionally more favorable among students with normal BMI living in rural areas or smaller towns. Students with abnormal body weight were generally unable to accurately assess their nutritional knowledge and dietary behaviors. These findings underscore the need for targeted nutrition education programs that strengthen both knowledge and practical skills. Future interventions should adopt a comprehensive approach that integrates nutrition education with practical food-related skills, self-regulation, informed food decision-making, and supportive food environments, rather than relying solely on traditional information-based education.

## Figures and Tables

**Table 1:** Characteristics of the studied group of students according to BMI (N = 1440).

Factor	Categories	BMI			Chi2 Test
		Underweight N = 153	Normal N = 1016	Overweight/ Obesity N = 271	
Sex (%)	girls	76	52	29	<0.001
	boys	24	48	71	
Age (years)	x ± sd	14.4 ± 2.8	14.5 ± 2.6	14.0 ± 2.6	<0.001 **
	Me	15.0	15.0	14.0	
	min-max	10–18	10–18	10–18	
Age (%)	10–12 y.	31	29	34	0.009
	13–15 y.	25	27	34	
	16–18 y.	44	44	32	
Place of residence (%)	village	33	35	37	0.255
	Town < 50,000	14	19	21	
	city > 50,000	53	46	42	
Education level of the mother/legal guardian (%)	primary	16	10	14	0.028
	secondary	30	31	36	
	higher	54	59	50	
Physical activity (%)	low	17	23	25	0.159
	moderate	54	47	42	
	high	29	30	33	
NRK * (%)	insufficient	11	9	16	<0.001
	sufficient	35	36	44	
	good	45	42	36	
	very good	9	13	4	
Nutritional knowledge self-assessment (%)	insufficient	9	9	10	0.036
	sufficient	25	35	39	
	good	48	42	43	
	very good	18	14	8	
NRK and Nutritional knowledge self-assessment comparison ***		0.007	0.705	<0.001	
NRP * (%)	insufficient	40	42	51	0.036
	sufficient	52	51	45	
	good	8	7	4	
	very good	0	0	0	
Nutritional practice self-assessment (%)	insufficient	3	4	4	0.012
	sufficient	16	17	26	
	good	64	66	62	
	very good	17	13	8	
NRP and Nutritional practice self-assessment comparison ***		<0.001	<0.001	<0.001	

* Scale 0–12 points—insufficient (0–3 pkt.), sufficient (4–6 pkt.), good (7–9), very good (10–12 pkt.); ** Kruskal–Wallis test.; *** Wilcoxon test.

**Table 2:** Assessment of students’ NRK and NRP according to BMI (N = 1440).

**Questions**	**BMI**			**Chi2** **Test**	**BMI**	
	**Underweight** **N = 153**	**Normal** **N = 1016**	**Overweight/** **Obesity** **N = 271**		**Underweight** **N = 153**	**Overweight/** **Obesity** **N = 271**
**NRK**	% of correct answers				OR ** (ref. BMI normal) (95% CI) *p*-value	
Q1: How many meals should children and adolescents eat a day?	62	59	48	0.002	1.117 (0.787–1.585) 0.534	0.638 (0.488–0.835) 0.001
Q2: Which meal eaten regularly is particularly important for well-being at school?	88	86	77	0.001	1.165 (0.698–1.943) 0.559	0.545 (0.391–0.760) <0.001
Q3: How often should children and adolescents eat fruit and vegetables?	59	59	46	0.001	0.974 (0.690–1.377) 0.883	0.593 (0.453–0.776) <0.001
Q4: Which products contain more dietary fiber?	65	64	49	<0.001	1.037 (0.727–1.479) 0.842	0.545 (0.416–0.714) <0.001
Q5: How many servings of milk and/or dairy products should children and adolescents consume a day?	18 ^A^****	16 ^A^	18 ^A^	0.729	1.155 (0.742–1.798) 0.522	1.110 (0.779–1.581) 0.562
Q6: Which type of meat and/or meat products are most recommended for consumption?	76 ^B^	77	68	0.018	0.994 (0.666–1.484) 0.977	0.658 (0.490–0.883) 0.005
Q7: How often should children and adolescents eat sea fish?	9 ^C^	22	20	0.001	0.358 (0.203–0.633) <0.001	0.885 (0.634–1.235) 0.472
Q8: What is at the top of the Healthy Nutrition and Lifestyle Pyramid?	33	33	31	0.809	1.000 (0.696–1.437) 0.999	0.910 (0.681–1.215) 0.521
Q9: Which products should replace sweets?	78	74	65	0.004	1.270 (0.843–1.914) 0.253	0.658 (0.494–0.876) 0.004
Q10: Which products contain a lot of salt?	73	82	73	0.001	0.612 (0.414–0.906) 0.014	0.597 (0.437–0.814) 0.001
Q11: What is the recommended amount of water consumption for children and adolescents?	56	59	56	0.433	0.856 (0.608–1.206) 0.374	0.862 (0.658–1.129) 0.281
Q12: What is the base of the Healthy Nutrition and Lifestyle Pyramid?	41	40	34	0.184	1.007 (0.712–1.424) 0.968	0.772 (0.583–1.022) 0.071
x ± sd *	6.2 ± 2.2	6.3 ± 2.2	5.5 ± 2.2	<0.001 ***		
Me	6.0	7.0	5.0			
min-max	1–11	0–11	0–11			
**NRP**	% of correct answers			Chi2 test	OR ** (ref. BMI normal) (95% CI) *p*-value	
Q1: How many meals a day do you usually eat?	25	28	27	0.812	0.882 (0.598–1.300) 0.526	0.968 (0.717–1.307) 0.833
Q2: Do you eat breakfast in the morning before going to school?	54	51	41	0.004	1.135 (0.807–1.597) 0.465	0.654 (0.499–0.859) 0.002
Q3: How often do you eat fruit and vegetables?	37	33	22	0.001	1.189 (0.835–1.695) 0.337	0.586 (0.427–0.803) 0.001
Q4: How often do you eat whole grain products?	18	14	12	0.178	1.390 (0.889–2.173) 0.148	0.831 (0.552–1.251) 0.375
Q5: How many servings of milk and/or dairy products do you consume per day?	11 ^A^	14 ^A^	15 ^A^	0.568	0.782 (0.458–1.335) 0.368	1.083 (0.741–1.585) 0.679
Q6: Which type of meat and/or meat products do you eat most often?	70 ^B^	62	48	<0.001	1.402 (0.970–2.025) 0.072	0.564 (0.430–0.739) <0.001
Q7: How often do you eat fish?	8 ^C^	8	7	0.949	0.982 (0.522–1.847) 0.956	0.920 (0.553–1.530) 0.747
Q8: What fat are the foods you eat fried in?	56	55	58	0.676	1.010 (0.717–1.422) 0.955	1.130 (0.861–1.482) 0.379
Q9: How often do you eat sweets?	3	4	5	0.694	0.846 (0.328–2.182) 0.730	1.262 (0.664–2.400) 0.477
Q10: How often do you eat salty snacks?	8	6	4	0.316	1.225 (0.646–2.323) 0.534	0.667 (0.355–1.252) 0.208
Q11: How much water do you drink a day?	40	45	47	0.373	0.814 (0.576–1.151) 0.245	1.083 (0.828–1.417) 0.560
Q12: What is your physical activity?	80	72	69	0.048	1.519 (1.001–2.307) 0.049	0.845 (0.632–1.130) 0.256
x ± sd *	4.1 ± 1.7	3.9 ± 1.7	3.6 ± 1.6	0.007 ***		
Me	4.0	4.0	3.0			
min-max	1–9	0–9	0–8			
Wilcoxon effect size NRK/NRP	0.66	0.73	0.67			

OR, odds ratio; * Scale 0–12 points; ** Wald test; *** Kruskal–Wallis test, **** Wilcoxon test; QNRK-QNRP pairs sharing the same letter (A, B, or C) were not significantly different within a given BMI category (*p* > 0.05).

**Table 3:** Relationships between nutritional knowledge and nutritional practice in the studied group of students (N = 1440).

Relationship	NRP ***	Knowledge Self-Assessment	Practice Self-Assessment
BMI			
underweight			
NRK *	0.206 **	0.144	0.083
NRP *		0.154	0.299 ***
Knowledge self-assessment			0.684 ***
normal			
NRK	0.376 ***	0.234 ***	0.137 ***
NRP		0.235 ***	0.304 ***
Knowledge self-assessment			0.460 ***
overweight/obesity			
NRK	0.295 ***	0.073	0.049
NRP		0.245 ***	0.183 ***
Knowledge self-assessment			0.429 ***

* Scale 0–12 points; ** Spearman rank correlation significant at the 0.05 level (two-tailed); *** Spearman rank correlation significant at the 0.01 level (two-tailed).

**Table 4:** Association of socio-demographic and lifestyle factors with the students’ NRK and NRP according to BMI (N = 1440).

Factor				BMI					
	Underweight N = 153			Normal N = 1016			Overweight/Obesity N = 271		
	Unstandardized B (95% CI)	Standardized β	*p*-Value	Unstandardized B (95% CI)	Standardized β	*p*-Value	Unstandardized B (95% CI)	Standardized β	*p*-Value
NRK *									
Sex	−0.808 (−1.540; −0.075)	−0.159	0.031	−0.607 (−0.865; −0.350)	−0.137	<0.001	−0.770 (−1.321; −0.219)	−0.161	0.006
Age	0.215 (0.100; 0.330)	0.277	<0.001	0.116 (0.063; 0.169)	0.138	<0.001	0.187 (0.082; 0.293)	0.220	0.001
Place of residence	0.267 (−0.083; 0.618)	0.112	0.133	0.103 (−0.051; 0.257)	0.042	0.190	0.387 (0.086; 0.688)	0.158	0.012
Education level of the mother/legal guardian	0.823 (0.406; 1.240)	0.286	<0.001	0.773 (0.578; 0.968)	0.236	<0.001	0.076 (−0.281; 0.434)	0.025	0.675
NRP *									
Sex	−0.124 (−0.698; 0.450)	−0.032	0.669	−0.120 (−0.305; 0.065)	−0.035	0.202	−0.256 (−0.639; 0.127)	−0.072	0.189
Age	−0.080 (−0.178; 0.019)	−0.134	0.111	−0.006 (−0.044; 0.032)	−0.009	0.758	−0.051 (−0.124; 0.022)	−0.080	0.171
Place of residence	0.214 (−0.060; 0.489)	0.116	0.124	−0.125 (−0.232; −0.017)	−0.065	0.023	0.184 (−0.021; 0.390)	0.101	0.079
Education level of the mother/legal guardian	0.107 (−0.235; 0.449)	0.048	0.537	0.055 (−0.085; 0.195)	0.021	0.444	−0.048 (−0.294; 0.198)	−0.021	0.701
Level of physical activity	0.930 (0.548; 1.311)	0.374	<0.001	0.868 (0.739; 0.996)	0.366	<0.001	0.961 (0.733; 1.189)	0.452	<0.001
NRK *	0.210 (0.085; 0.335)	0.272	0.001	0.268 (0.224; 0.312)	0.345	<0.001	0.211 (0.130; 0.293)	0.284	<0.001

* Scale 0–12 points; NRK Linear regression: BMI underweight: adjusted R2 = 0.218, F = 11.580, *p* < 0.001; BMI normal: adjusted R2 = 0.111, F = 32.751, *p* < 0.001; BMI overweight/obesity: adjusted R2 = 0.109, F = 9.240, *p* < 0.001; NRP Linear regression: BMI underweight: adjusted R2 = 0.220, F = 8.160, *p* < 0.001; BMI normal: adjusted R2 = 0.284, F = 67.948, *p* < 0.001; BMI overweight/obesity: adjusted R2 = 0.268, F = 17.451, *p* < 0.001.

## Data Availability

The dataset used and/or analyzed during this study is available from the corresponding author upon reasonable request. The data are not publicly available due to ethical restrictions and participant confidentiality.
